# Use of Digital Media for Home-Based Sports Activities during the COVID-19 Pandemic: Results from the German SPOVID Survey

**DOI:** 10.3390/ijerph18094409

**Published:** 2021-04-21

**Authors:** Michael Mutz, Johannes Müller, Anne K. Reimers

**Affiliations:** 1 Institute of Sport Science, Justus-Liebig-University Giessen, Kugelberg 62, 35394 Giessen, Germany; johannes.mueller@sport.uni-giessen.de; 2 Department of Sport Science and Sport, Friedrich-Alexander-University Erlangen-Nuremberg, Gebbertstrasse 123b, 91058 Erlangen, Germany; anne.reimers@fau.de

**Keywords:** COVID-19, online fitness, digitalization, physical activity, social inequality

## Abstract

Health authorities recommend digital tools for home-based sport and exercise routines to stay active and healthy during the COVID-19 pandemic. The study investigates the prevalence, duration, most popular activities, and social selectivity of home-based digital sport and its contribution to overall levels of sporting activity during the pandemic. It is based on cross-sectional survey data (*n* = 1508), representing the population >14 years living in Germany. Data collection took place in October 2020, using computer-assisted web interviewing. Results show that overall, 23% of respondents used digital media for sports activities at least one time during the COVID-19 pandemic. Numbers increased during the lockdown and decreased afterwards. People engaged in a variety of fitness workouts, most frequently practiced with the help of publicly accessible fitness videos from video-sharing platforms. Digital sports practitioners are younger, better educated, and financially better off. Females are overrepresented. Individuals engaged in digital sports achieved 30 min/week more sports activity during the pandemic compared to individuals solely involved in offline sports. Hence, home-based digital sports activities were a popular means to stay active, particularly in the period of the lockdown. Strong social disparities indicate that the possible health benefits of digital sports only reach out to particular population groups.

## 1. Introduction

Digital technologies have transformed social life in profound ways during the last decade, not omitting sports activities and health behaviors. Apps and wearables monitor and compare physical activity, allow for self-tracking pursuits, and connect with online communities [1,2,3]. Online fitness clubs and influencers enable their followers and members to engage in fitness activities nearly anywhere and anytime, while social media platforms showcase lifestyle sports and fitness trends and distribute health- and fitness-related messages [4]. Many accounts elaborate on experiences with using fitness and physical activity apps or wearable devices [5,6,7,8,9,10,11,12]. Others assess the contribution of these technologies for physical activity levels [13,14,15] or explore gender and age differences in the usage of fitness apps [16]. Qualitative studies explored how users of apps and wearables engage with respective data, e.g., for self-surveillance and self-presentation purposes, or for reassuring themselves as productive and active agents for health and wellbeing [17,18,19]. Despite the fact that a large group of individuals still plays sport and exercises solely offline, the ‘digital age’ and processes of mediatization have clearly left their mark in the sport and fitness sector [20,21].

This paper addresses the use of digital sports activities at home. Digital sports activities are conceptualized as all sports and exercise activities that are essentially supported by or make use of digital media or digital technologies, like live-streamed exercises, on-demand videos, DVDs, or Smartphone apps. A particular catalyst for home-based digital sport activities was the COVID-19 pandemic. When containment and mitigation policies containing the spread of the virus forced governments worldwide to lock down non-essential public infrastructure, the sport sector was often the first to be closed and the last to be re-opened. Regarding Germany, for instance, sports facilities were closed in a first lockdown from late March to late May 2020 (depending on Federal State regulations), and again from November 2020 throughout the winter season. Hence, fitness studios, sports clubs, swimming pools, and other public facilities could not be approached during these periods. Findings based on representative population samples indicate that these policies led to a strong decline in sports activity in Germany, both in adults [22] as well as children and youths [23]. However, the same studies suggest that the level of light and moderate physical activity (e.g., going for walks, playing outdoors) has increased. 

Many regular users and members of sports clubs and fitness studios were searching for alternatives, namely individualized, home-based and, presumably, digital sports activities. Health experts advise people on how to stay active during the pandemic, thereby recommending digital tools for home-based routines [24,25,26]. Likewise, the World Health Organization [27] recommends the use of “online resources” for maintaining a basic physical activity level during the pandemic. 

Global surveys indicate that professionals in the health and fitness sector regard “online fitness” as the most important fitness trend in 2021 [28]. However, reliable data based on representative population surveys on the use of digital sports activities during the pandemic are still missing. Weekly surveys in April and May 2020 show that 19–23% of British adults were engaged in online fitness activities during the COVID-19-related lockdown [29]. A German study indicates that roughly 2% of adults started with online fitness activities in the first two weeks of the German lockdown [22], but this share may have increased in later stages of the pandemic. A recent study from Belgium found that individuals who were used to exercising with online support before the lockdown were more likely to increase their exercise levels during the lockdown [30]. Studies based on convenience samples also present data on sports activities during the lockdown, but respective findings do not allow for general conclusions. An online survey in Brazil, for instance, indicates that 23% of the respondents engaged in online fitness classes and 20% watched fitness and exercise videos from the internet during the pandemic [31]. These surprisingly high numbers in this study, however, may be due to the distribution of the questionnaire via WhatsApp and Facebook, thus to internet-affine respondents only. 

However, many key questions regarding the use of digital sports at home are still unsolved, for instance, the question on the prevalence and contents of digital sports, the user’s socio-demographic characteristics, as well as digital sports’ contribution to a person’s overall activity level. Thus, the aims of the study are to investigate (1) the proportion of people engaging in digital sports activities at home, (2) the sources and materials that are used for practicing sports digitally, (3) the most popular exercises and practices, (4) the socio-demographic characteristics of people engaging in digital sports activities, and (5) the contribution of digital sports activities to levels of overall sports activity. This study puts these questions in relation to the COVID-19 pandemic, giving first clues on how the pandemic might have changed the sporting landscape, presumably by pushing people into digital alternatives to traditional offline sports. 

## 2. Materials and Methods

### 2.1. Study Design and Procedure

The present study “Examining Physical Activity and Sports Behavior in the Face of COVID-19 Pandemic” (SPOVID) is based on a large-scale, cross-sectional survey design. A sample, representing the German population (>14 years), was questioned using computer-assisted web interviewing (CAWI). The sample represents the German population according to age (*M* = 48.8; *SD* = 18.6), gender (49% males, 51% females), educational level (27% lower, 32% medium, and 41% upper secondary degree) and residency in the 16 German Federal States.

The survey was integrated into FORSA Omninet, an existing nation-wide online panel. FORSA is among the leading organizations in public opinion polling in Germany, able to provide timely, reliable, and representative public opinion data. A specific feature of this panel is that panel recruitment takes place solely offline via telephone surveys which make use of random digit dialing procedures. Thus, the online panel also represents those population groups adequately who have a greater distance to internet services. Initial recruitment procedures were in line with all legal requirements as well as the guidelines of the Association of German Market and Social Research Institutes for online surveys and interviews with minors. All participants provided written consent to be contacted via email for online surveys and they took part in each survey voluntarily.

Data collection took place from 16 October to 3 November 2020. Regarding Germany, sports infrastructure closed on 1 November 2020 due to an accelerated spread of the Coronavirus. Hence, this survey took place right before the second nationwide lockdown of sports and leisure facilities. Respondents were invited via email to participate in the survey and were able to answer the survey on their computer, tablet, or mobile phone. Overall, 1508 individuals completed the survey and were included into this analysis. All participants gave informed consent to participate in this study and the study procedures received approval from the ethics commission of the Friedrich-Alexander-University Erlangen-Nuremberg (Reg. 387_20B). 

### 2.2. Measures 

Digital sports activities. We assessed digital sports activities with several questions: A first screening question identified those who were engaged in any digital sports activity in the time since the beginning of the COVID-19 pandemic in Germany (Please think of the last six months: Have you used videos, online programs, or other digital media for sports activities at home?). Those respondents who replied with ‘yes’ then received a second, open-ended question that inquired a short description of the types of activities or exercises that were performed (Which types of sport or exercises did you practice when using digital media: Please describe these activities in a few sentences or keywords!). A third question then addressed the duration per week that respondents spent with digital sports activities (How many hours did you use videos, online programs, or other digital media for sports activities at home?). We asked this question with regard to three periods: (a) before the pandemic, (b) during the April–May lockdown and (c) in the last week, i.e., a week in October 2020 when sports facilities were open, but the COVID-19 incidence was already on the rise again in Germany. Respondents indicated their answers on a rating scale with eight answer categories: (1) did not exercise or play sports; (2) less than 1 h; (3) about 1 h; (4) about 2 h; (5) about 3–4 h; (6) about 5–6 h; (7) about 7–14 h; (8) 15 h or more. We recoded the answers to calculate the weekly duration of digital sports activities.

Overall level of sports activity. During an initial question of the survey, respondents were asked to indicate the time spent with sport and exercise activities in hours per week (How much time did you play sport or exercise in your leisure time). Respondents answered this question with reference to (a) the time before pandemic and (b) the last week. The same rating scale with eight answer categories was provided (see above), ranging from (1) did not exercise or play sports to (8) 15 h or more. Again, we recoded values for capturing weekly hours of sports activities. 

Socio-demographic variables. To assess socio-economic and socio-demographic correlates of digital sports activities, we used several variables that all correlate with participation in (traditional offline) sports in Germany [32,33,34]: age (in years), gender, educational level (measured by school-leaving qualifications), net income (in ten categories from ‘no income’ up to ‘>5000 EUR/month’), immigrant status (1st and 2nd generation immigrants) and residency in an urban or rural area (measured by place of residence’s total population). 

### 2.3. Coding of Open-Ended Question on the Types of Digital Sports Activities

The open-ended question was analyzed based on a quantitative content analysis. Two coders thoroughly read all answers and developed a coding scheme for eight main categories of answers and one residual category. The answers were then coded by one author and one student assistant according to the coding scheme (κ = 0.91), thereby assigning responses to categories. After correcting for coding disagreements, we calculated the share of respondents whose open answer fitted into a specific answer category to identify the most popular categories. 

### 2.4. Statistical Analyses

To accommodate the purpose of descriptive statistics, we report the share of people engaged in any digital sports activity and in the most popular activity categories. Using logistic regression analyses, we document associations between participation in digital sport and socio-economic as well as socio-demographic variables. To accommodate comparison reasons, we calculate the same logistic regression model with ‘offline’ sports engagement as the dependent variable. To be able to compare regression estimates, we use inactive respondents as the reference group in both models. Finally, we apply Generalized Linear Models (GLM) to estimate the contribution of digital sports to overall levels of sports activity. The GLM models include age, gender, education, net income, immigrant status, and urban/rural residency as covariates. A level of 0.05 was set as a threshold to determine statistical significance. All analyses were conducted with IBM SPSS 25 (IBM Corporation, Armonk, NY, USA).

## 3. Results

### 3.1. Prevalence of Digital Sports Activities 

Considering all 1508 respondents, 23% reported as having used digital media for sports activities for at least one time during the COVID-19 pandemic. During the pre-pandemic period, only 12% were involved in respective activities. During the lockdown of sports infrastructure in April 2020, the share of digital sports practitioners increased considerably to 19%. However, not everyone who tried digital sports have maintained these activities. Many have apparently only switched to digital sports during the first lockdown during April–May 2020, when public and private sports infrastructure closed. During October 2020, when sports facilities had re-opened again, the share of digital sports practitioners decreased to 14%, hence almost to the pre-pandemic level.

### 3.2. Time Spent with Digital Sports Activities

Those who were actively engaged in digital sports activities in the pre-pandemic period, spent on average 98 min per week (*SD* = 120) with these activities. Time spent in digital sports activities increased to 131 min per person and week (*SD* = 130) in April and May 2020, when sports infrastructure was locked down. It finally consolidated at 117 min per week (*SD* = 139) in October, when sports infrastructure was open again. These numbers indicate that the average active user of digital sports increased their engagement when public sports infrastructure was not available and reduced it when facilities re-opened after the lockdown.

### 3.3. Media and Technologies Used for Digital Sports Activities

Active users of digital sports used a variety of media and technologies for their activities. The largest proportion watched publicly accessible fitness videos from online video-sharing platforms like YouTube (66%) or received video material from their sports clubs or fitness studios (15%). Another 9% of active users practiced with the help of DVDs, hence with offline video content. Taken together, a very large share of users engaged in time-independent, video-supported exercises. Additionally, a share of 10% took part in live streamed courses, i.e., where participants joined a scheduled online meeting to exercise virtually together. Hence, this format may give users the impression of participating in a social activity and being part of an (imagined) group.

### 3.4. Most Popular Digital Sports Activities

Digital sports is a rather blanket term including a broad range of activities and practices. To get a deeper insight into the types of activities practiced with digital media and technologies, we used an open-ended question format. Overall, 96% of all respondents engaged in digital sports activities gave a valid answer to the open-ended question that helped to identify one or more particular digital sports activities. Most respondents indicated one activity, but some referred to more activities. Overall, 525 valid responses from 334 respondents were identified. After independent coding of all responses by two researchers, eight main categories and one residual category (for all answers not assigned to a main category) emerged (Table 1).

A proportion of 34% mentioned general fitness activities, for instance, full body training, high intensity training, circuit training or made reference to online fitness platforms with varied courses (e.g., Gymondo or LesMills) or YouTube channels that offer mixed workouts (e.g., Pamela Reif, Madfit, Sascha Huber). Strength and weight training is another popular category: 29% engaged in these exercises, however, significantly more men than women (Χ^2^_1_ = 19.3; φ = 0.24; *p* < 0.01). This category combines strength training with specific equipment (dumbbells or TRX slings) and respective exercises with a person’s own body weight, like Push Ups or Sit Ups. Having similar popularity are body and mind practices, a category that summarizes practices like Yoga, Tai Chi or Pilates. Considering all respondents, 28% engage in one of these practices, among them significantly more women than men (Χ^2^_1_ = 28.1; φ = 0.29; *p* < 0.01). Gymnastic is a category that includes general gymnastic and stretching exercises as well as functional gymnastics (e.g., pregnancy gymnastics): 20% of active users mentioned such activities. Another 17% referred to body shaping or body modeling workouts. Frequently mentioned here were workouts with a focus on legs, glutes, special abdominal muscle training or workouts using power plates. A significantly higher share of women than men engaged in these workouts (Χ^2^_1_ = 14.1; φ = 0.21; *p* < 0.01). Endurance training also is mentioned by 17% of digital sports practitioners. This category includes training and exercise with spinning bikes, ergometers, or treadmills, as well as endurance and cardio training at home without specific equipment. Therapeutic and health-related exercises, mentioned by 12%, is another category that summarizes therapeutic and preventive exercises for the elderly, or recovery training with a health-related goal. Most of the answers in this category refer to back training or exercises to reduce back pain. A final category refers to dance and dance-based workouts, respectively. This category includes ‘classic’ dance courses (Latin, Salsa, Hip Hop etc.) and rhythmic workouts that include dance steps and moves (e.g., Zumba). Overall, 5% of the active users of digital sports mentioned a dance activity, with women being overrepresented in this group (Χ^2^_1_ = 4.2; φ = 0.11; *p*= 0.04).

Overall, these descriptions indicate that digital sports activities were predominantly fitness exercises and workouts. Only very few traditional sports were mentioned (e.g., Jiu Jitsu, Golf, Boxing) that were practiced with the help of digital media during the pandemic. Moreover, the findings suggest that exergames, i.e., video games that include physical activity, are either not practiced by many or not associated with the terms “sport” and “exercise”. As both traditional sports and exergames are only mentioned sparsely, they were assigned to the residual category.

### 3.5. Social Inequality of Online Sports Participation 

Social stratification of sport is widely discussed, and recent German studies indicate that gender, age, immigration status, and social class influence overall participation in sport [32,33,34]. However, digital sports activities have not been studied yet within a social inequality framework.

Logistic regression analyses with participation in any digital sports activity (vs. being inactive) reveal that these sports activities are highly selective, and users differ in various socio-economic and socio-demographic characteristics (Table 2). Users of digital sports activities are of younger age (OR = 0.95, *p* < 0.001). Females are highly overrepresented in this group (OR = 1.70, *p* = 0.007) as well as individuals with higher educational levels (OR = 2.91, *p* < 0.001). Moreover, active users of digital sports have a higher net income (OR = 1.24, *p* = 0.001). Hence, digital sports attract socially privileged individuals, who are younger, better educated, and financially better off. 

Participation in traditional offline sports activities also is socially selective, however to a lesser degree. Engagement in offline sports (vs. being inactive) is associated with younger age (OR = 0.95, *p* < 0.001) and higher educational (OR = 1.56, *p* = 0.018) and income levels (OR = 1.18, *p* = 0.001), but these effects are less pronounced compared to digital sports activities. To contrast to digital sports, no gender effect is found for participation in offline sports. Overall, the higher social selectivity of digital sports also is indicated by the Pseudo-R^2^ of 0.343, which is substantially higher compared to 0.077 in the model for offline sport participation.

### 3.6. Contribution of Digital Sports Activities to Levels of Overall Sports Activity

To assess whether or not digital sports activities relate to levels of overall sports activity, Generalized Linear Models (GLM) are applied, with general sport activity as the dependent variable. Involvement in online and offline sport activities is used as a predictor (three categories: active including digital sports vs. active in offline sports only vs. inactive) and age, gender, education, net income, immigrant status, and urban/rural residency as covariates. 

Results reveal that the group of individuals who used digital sports activities during the pandemic stands out with an above-average “normal”, i.e., pre-pandemic, sports activity level (M = 176 min/week, *SE* = 10). Those who were solely engaged in offline sports during the pandemic had a significantly lower pre-pandemic activity level (M = 137 min/week, *SE* = 7). Likewise, those who were inactive during the pandemic reported a very low activity level in the time before the COVID-19 outbreak (M = 19 min/week, *SE* = 10). The mean pre-pandemic level of sporting activity is significantly higher in the active group with digital sports activities compared to the active group with offline activities only (F_1__,1142_ = 11.3; *p* = 0.001) as well as the inactive group (F_1__,665_ = 173.5; *p* < 0.001). Hence, digital sports activities appeal to those with a strong affinity to being active, i.e., who usually spent a significant amount of leisure time with sports and exercise.

Moreover, digital sport activities help individuals to maintain a relatively high level of sports activity during the COVID-19 pandemic. The digitally active group spent significantly more time per week in October 2020 with sport and exercise (M = 149 min/week, *SE* = 10) compared to those who engaged in offline sports only (M = 119 min/week, *SE* = 7). The mean difference between these two groups is significant (F_1__,1144_ = 7.3; *p* = 0.007). Digital sports activities, thus, help people to stay active when the pursuit of traditional offline sports activities was restricted. Unsurprisingly, the inactive group reported almost no sports activity in October 2020 (M = 4 min/week, *SE* = 10) and, thus, has a significantly lower sports activity level during the pandemic than those who were involved in digital sporting practices (F_1__,665_ = 212.1; *p* < 0.001).

**Figure 1:**
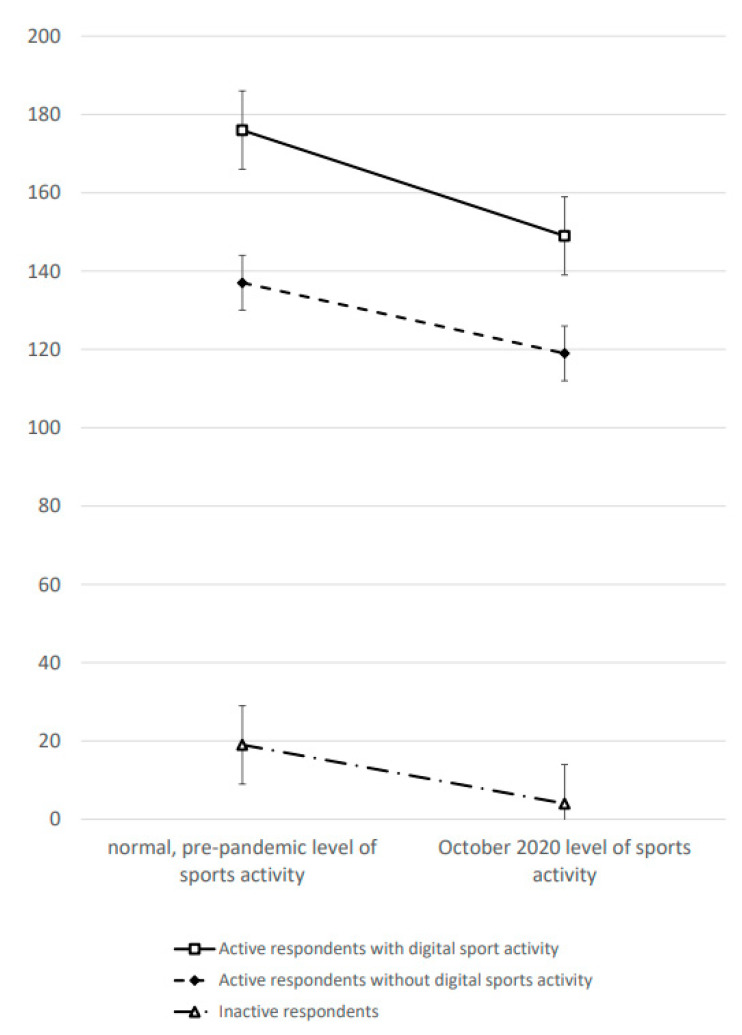
Level of sports activity (min/week) in the pre-pandemic period and in October 2020. Note: SPOVID Study. Estimated marginal means based on Generalized Linear Models. Error bars show the standard error of the mean. Differences in activity levels between active respondents with digital sport activity and active respondents without a digital sport activity are significant with *p* < 0.001. The time-group interaction is insignificant (*p* = 0.504).

Comparisons of responses with reference to “normal” (pre-pandemic) activity levels and with reference to October 2020 (during the pandemic) reveal a decline in all three groups (Figure 1). Hence, sport and exercise activities were decreasing during the COVID-19 pandemic. This difference amounts to −27 min/week among active respondents with an involvement in digital sports activities, −19 min/week among active respondents without involvement in digital sports activities, and −15 min/week among inactive respondents. There are no statistically significant differences in these changes between the three groups, i.e., the time–group-interaction is insignificant (F_2__,1479_ = 0.7; *p* = 0.504). Hence, an involvement or intensification of digital sports activities cannot protect against the general decrease of sports activity during the pandemic.

## 4. Discussion

### 4.1. Principal Findings

Based on representative survey data collected during the period of the COVID-19 pandemic in Germany (October–November 2020), this study provides some key insights regarding digital sports activities. Regarding the number of active users, this analysis revealed that every fifth German (>14 years) has used digital media for sports during the lockdown. This is exactly the share of the population as reported in British surveys for the UK [29]. During a pandemic, where population levels of sport and exercise generally declined in many countries [22,35,36,37,38], digital sports became more popular. 

Results further show that general as well as specific strength-, endurance-, body modelling- and dance-based workouts represent the most popular fitness exercises practiced during the pandemic. However, these workouts capture only a very small segment of the sporting landscape. Accompanying the reopening of sports clubs and fitness centers, the use of digital sports decreased again. Sport club representatives fear that the pandemic will lead to a substantial loss of members and existence-threatening situations [39]. However, digital sports do not appear to be a main driver for such a scenario: Concerning many, digital sports were welcome as a workaround in a period where public facilities were closed, but findings do not suggest that this resulted in a general shift from offline to online sports. 

Home-based digital sports require technological devices, sports equipment, certain sports competence, and space at home. Against this background, it is comprehensible that digital sport is socially more selective than traditional offline sport. Ng et al. [40] have shown that fitness apps and wearables are more popular among youths from affluent families. This study extends existing knowledge by showing that practitioners of digital sports are younger, better educated, and financially better off. Particularly the elderly participate only rarely in digital sports. Similar findings were reported for the use of health and fitness apps [41]. The underrepresentation of the elderly among digital sports users is presumably due to technological barriers [42] or a lack of motivating and meaningful health-oriented content within digital sports programs that is appropriate for older subjects [43]. Moreover, digital sports attract more females than males. Particularly, body shaping- and dance-based workouts, as well as body and mind practices like yoga, reach out to a majority of women. Scholars have often pointed to an underrepresentation of girls and women in German sports clubs [33,44], but fitness activities constitute a more gender-equal field in the sporting landscape [32]. 

Digital sports contribute to the general level of sports activity. During the pandemic, active users of digital sports accumulated an additional time in sports activities of 30 min per week, on average. Hence, digital sports support individuals to stay active and, thus, can have an important function for public health during the pandemic, supporting respective claims of experts and public health authorities [25,26,27]. However, data on the pre-pandemic level of sports activity of active users of digital sports show that this group already was more active before the COVID-19 crisis. Thus, it can be assumed that digital sports helped active people to stay active during the pandemic but did not motivate larger shares of inactive people to start with (digital) sports activities during the lockdown. 

### 4.2. Theoretical and Practical Implications

Taking a conceptual, theoretical, and practical level, this study comes with several implications. First, it helps clarify the concept and common understanding of ‘digital sports’. The operational definition used here equated digital sport with all sporting and exercise activities that are essentially supported by or make use of digital media or digital technologies. Responses indicate that people are more likely to subsume video-based activities under this term, i.e., where concrete exercises and tasks are shown (either live or recorded). Simply using a Smartphone app that records or tracks data does not suffice to turn an ‘offline’ sporting activity into a ‘digital’ one. 

Second, the findings have wider implications with regard to the mediatization of society—a concept that recently has received substantial scientific attention [21,45,46,47]. Scholars in this field argue that an increasing number of face-to-face activities are substituted by mediated interactions, so that mediatized communication infiltrate and transform everyday life to an ever greater extent [48]. Although sport—as a bodily practice and social activity—is not easy to imagine as a purely media activity, it is still heavily influenced in its forms, contents, and organization by mediatization and digitalization. Hence, sport may be a prime example for the “moulding forces” [49] of (digital) media. 

Finally, practical implications may arise for sports clubs and gyms. Although data analyzed here does not indicate a long-lasting trend away from offline sports, voluntary sports clubs and commercial gyms nevertheless may be well-advised to incorporate digital activities permanently into their portfolio. These digital offers may extend traditional offline activities by providing possibilities for sports participation to individuals with limited time or limited mobility and particularly may attract female members and clients. Considering a post-pandemic world, it seems likely that few people will use digital sports activities as a complete substitute for offline sports, but for many this may become a temporary and easily accessible complement.

### 4.3. Strengths and Limitations

This study has strengths and limitations: Given the lack of studies that address the prevalence and social selectivity of digital sports, this research substantially adds to the state of knowledge on digital media supported sports. A major strength is the representative sample that allows for general conclusions on the German population (>14 years). Studies on sports activities in the pandemic rarely use representative data, but often build on convenience samples. However, data collection took place during October–November 2020, hence in one period during the pandemic. Supposedly, people adapt their behavior quickly, according to the dynamic of the outbreak. Hence, data on the use of digital sports from different periods (before, during, or after the pandemic) would be useful to better assess the general use, rise, and decline of digital sports, as well as specific individual and collective patterns of initiation and withdrawal. Moreover, questions on sports behavior during the lockdown during April and May 2020 were collected retrospectively, so memory gaps may affect responses. Finally, findings hold true for the German context, but may differ in other countries depending on the scope and strictness of containment policies put in force by national governments. 

## 5. Conclusions

This paper has shown that digital media play an important role for sports activities during the COVID-19 pandemic. A fifth of the German population (19%) engaged in digital fitness exercises at home during the lockdown. Thus, respective media and technologies helped people to stay active and healthy under the restrictions of COVID-19 mitigation policies. Findings suggest that digital sport was used as a workaround during the lockdown and many users switched back to traditional offline sports activities as soon as restrictions were suspended. Staying physically active is important during the pandemic situation and, particularly during lockdowns, sport offers by means of digital media are an important tool to achieve this public health goal [27]. However, a main challenge for future development of digital sports is the relatively high social selectivity. Particularly with regard to age and education, digital sports mirror the digital divide that pervades society in general [50]. The already existing inequalities with regard to access to and use of digital technologies have surely increased during the COVID-19 crisis (e.g., with remote work, distance learning, and digital sports) and findings presented here suggest that this digital gap also can impact physical activity and, in turn, health outcomes.

## Figures and Tables

**Table 1:** Categories of most popular digital sports activities.

Category	Description and Examples	Males (%)	Females (%)	Overall (%)
General fitness workout	General fitness exercises, e.g., full body training, circuit training, Tabata, HIIT, or reference to online fitness platforms with varied/mixed courses, e.g., Gymondo, LesMills	33.9	34.4	34.2
Strength and weight training ^a^	Muscle building and strength training, e.g., dumbbell training, Calisthenics, Body Pump, Push Ups, Sit Ups, CORE Training, suspension training with TRX slings	43.4	20.8	29.1
Body and mind practices ^b^	Practices that combine bodily movements or postures with breathing, concentration, alertness, or relaxation methods, e.g., Yoga, Pilates, Tai Chi, Qi Gong	11.6	38.8	28.3
(Functional) Gymnastic	Functional and general gymnastics and stretching exercises, including gymnastics and exercises during/after pregnancy	14.8	22.5	19.6
Body shaping/body modelling workouts ^b^	Body shaping and modelling workouts usually with reference to one or more body parts or body zones that a workout is focused on, e.g., legs, glutes and abdomen	6.6	22.6	16.9
Endurance training	Endurance training with or without equipment, e.g., use of ergometer, treadmill, spinning, cardio-workout	19.7	15.1	16.7
Therapeutic and health-related exercises	Therapeutic sports, preventive exercises for the elderly, recovery training, where respondents mentioned a specific illness or health-related goal, e.g., back training	9.8	13.2	11.9
Dance and dance-based workouts ^b^	Dance courses and dance-based workouts, e.g., Zumba, Aerobic, Salsa, Hip Hop	1.6	6.6	4.6
Unspecified/other	Residual category for all other responses that could not be assigned to one of the other categories, e.g., Exercise Games, Martial Arts	12.3	8.5	9.8

Note: ^a^ Men and ^b^ women significantly overrepresented, based on Chi^2^-Tests (*p* < 0.05).

**Table 2:** Logistic regression models for participation in online/offline sports vs. inactivity.

Participation in Online and Offline Sports	Online Sports vs. Inactive (*n* = 597)			Offline Sports only vs. Inactive (*n* = 1315)		
	*B*	*Exp(B)*	*p*	*B*	*Exp(B)*	*p*
Age (in years)	−0.05	0.95	<0.001	−0.02	0.98	<0.001
Gender (Ref. male)	0.53	1.70	0.007	0.00	1.00	0.996
Education - higher secondary - medium secondary (Ref. lower secondary)	1.07 0.21	2.91 1.24	<0.001 0.457	0.45 0.12	1.56 1.12	0.018 0.503
Net income (in 1000 €)	0.21	1.24	0.001	0.17	1.18	0.001
Immigrant Status (Ref. non-migrant)	0.46	1.58	0.127	0.29	1.33	0.218
Urban/rural residency - metropolitan area - small town/suburbs (Ref. rural area)	0.17 0.20	1.19 1.23	0.468 0.402	0.11 0.20	1.12 1.22	0.503 0.233
Pseudo-R^2^ (Nagelkerke)		0.343			0.077	

Note: SPOVID Survey. Logistic regression models. Table shows Logit coefficients (*B*), Odds Ratios (Exp[B]) and *p*-values.

## Data Availability

The data set of the SPOVID study is available from the first author upon request.
